# Impact of nutritional supplements on cognitive development of children in developing countries: A meta-analysis

**DOI:** 10.1038/s41598-017-11023-4

**Published:** 2017-09-06

**Authors:** Patrick Ip, Frederick Ka Wing Ho, Nirmala Rao, Jin Sun, Mary Eming Young, Chun Bong Chow, Winnie Tso, Kam Lun Hon

**Affiliations:** 1 Department of Paediatrics and Adolescent Medicine, The University of Hong Kong, Hong Kong, China; 2 Faculty of Education, The University of Hong Kong, Hong Kong, China; 3 Department of Early Childhood Education, The Education University of Hong Kong, Hong Kong, China; 4 China Development Research Foundation, Beijing, China; 5 Department of Paediatrics, The Chinese University of Hong Kong, Hong Kong, China

## Abstract

Nutritional supplements may be important on cognition but the evidence is heterogeneous. This meta-analysis aimed (1) to determine whether nutritional supplements provided to pregnant women or young children could improve cognitive development of children in developing countries, and (2) to explore how supplementation characteristics could improve children’s cognitive outcomes. This meta-analysis examined nutritional supplementation studies in 9 electronic databases and 13 specialist websites. Experimental studies were included if they were published from 1992 to 2016, were conducted in developing countries, had nutritional supplementation for pregnant women or children aged ≤8, and reported effect sizes on cognitive outcomes. Interventions with confounded components, such as stimulation and parenting, were excluded. 67 interventions (48 studies) for 29814 children from 20 developing countries were evaluated. Childhood nutritional supplementation could improve children’s cognitive development (d 0.08, 95% CI 0.03–0.13) and those with ≥5 nutrients was particularly beneficial (0.15, 0.08–0.22). Antenatal supplementation did not improve cognitive development (0.02, -0.01 to 0.06) except for those implemented in the first trimester (0.15, 0.03–0.28). In conclusion, childhood nutritional supplementation was beneficial to cognitive development but could be optimised by providing multiple nutrients; antenatal supplementation should target pregnancy women in the first trimester for better cognitive benefits.

## Introduction

Pregnant women and children under five are particularly vulnerable to micronutrient deficiencies (MND)^1^. As a response, the World Health Organization (WHO) has issued a series of guidelines on nutritional supplementation for pregnant women and young children^2^. Despite great efforts in the past decades, childhood undernutrition is still prevalent in developing countries^3^.

Nutritional supplements not only can improve growth and physical health of children in developing countries^4^, and are also important to their early development, including cognition^5–7^. The achievement of optimal early development is crucial, because it could reliably predict later health, education, and well-being^8, 9^.

Previous research suggests childhood supplementation that included iron and long-chain polyunsaturated fatty acids had minimal effects on cognitive performance^10, 11^, while supplementation with multiple micronutrients or adding multiple micronutrients to food appeared to have a benefit^12^. A recent meta-analysis has found a small but significant effect of nutritional supplements on child development^13^ and another systematic review reported that the combination of stimulation and nutritional supplementation benefited development^14^. However, the first meta-analysis did not analyse how intervention characteristics (e.g. types and quantities of supplements) may affect the cognitive benefits, and the second review did not provide any quantitative synthesis of data. Another meta-analysis investigated the benefits of nutrition interventions on cognitive development of children under two; this identified a small effect for postnatal intervention but a null effect for antenatal intervention^15^. Focusing on children under two allowed a more homogenous analysis, but the effect on higher-order cognitive performance, such as literacy and numeracy, cannot be studied. Furthermore, a Cochrane review has found that food supplementation’s effect on childhood cognitive development was mixed, while that for psychomotor development was medium sized, even though only two studies were synthesised^16^. This review has conducted a systematic subgroup analysis, which are useful for future intervention planning. Nevertheless, it only focused on food supplementation, limiting its applicability to nutritional supplementation in general.

The current meta-analysis combined a new systematic literature search (for articles in 2013–2016) with the review commissioned by Department for International Development, UK (DFID, for articles in 1992–2012) with the following aims: (1) to examine to what extent did pure nutritional supplementation (i.e. the only difference between the intervention and control arms was nutritional supplementation, but not stimulation, parenting, or cash transfer, etc.) improve cognitive development of young children in developing countries, and (2) to examine the influence of supplementation characteristics (i.e. timing of intervention, types and quantity of nutrients, and duration of follow-up) on intervention benefits.

## Results

### Study identification

**Figure 1:**
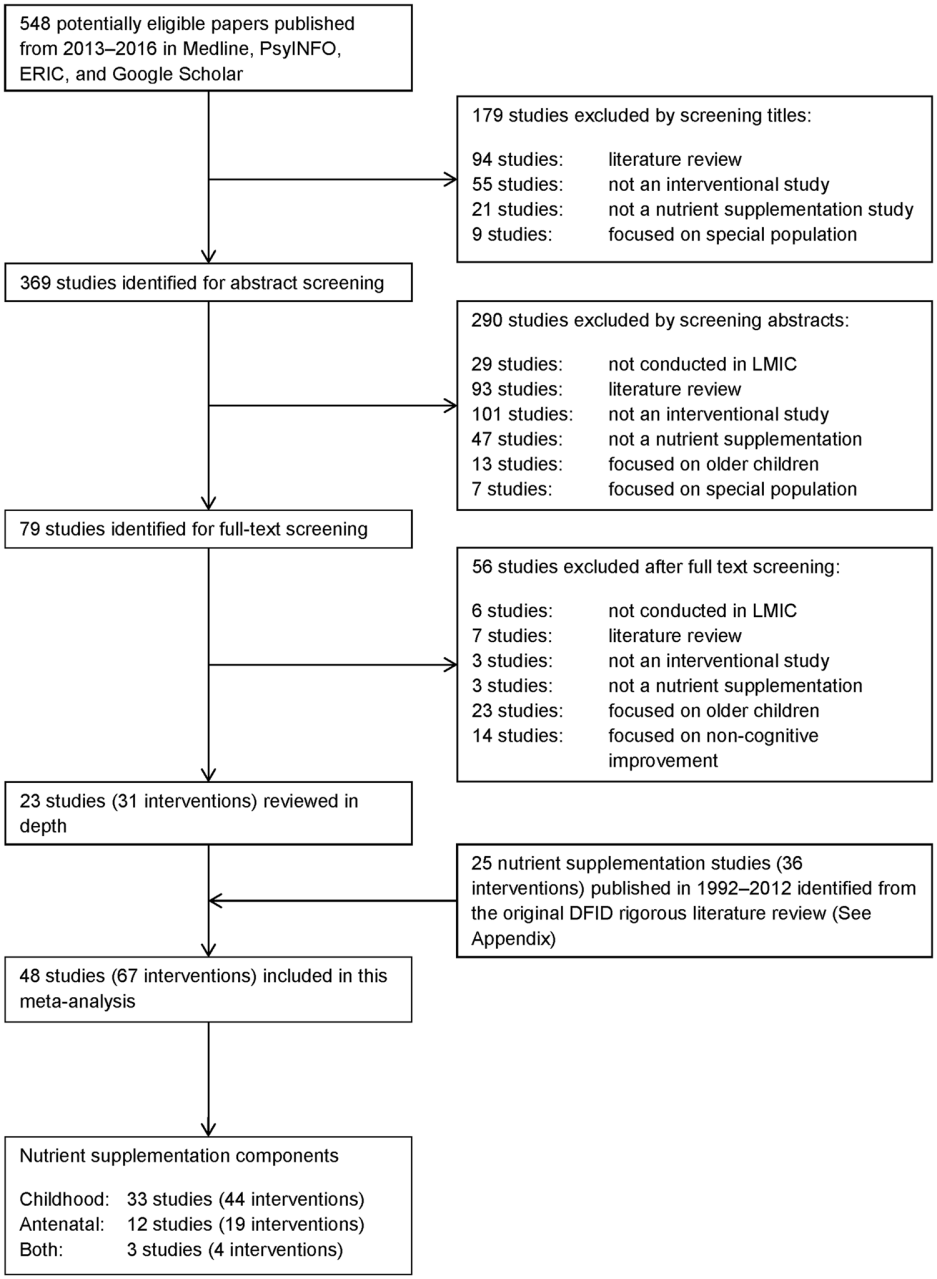
Identification of nutritional supplementation studies.

The original DFID study identified 25 studies published from 1992 to 2012. Details of the literature search of the DFID study can be found in the Appendix Fig. 1. The new literature search identified 548 studies published from 2013 to 2016. After iterative screening on study title, abstract, and full text, 525 studies were excluded, most commonly because they were observational/review studies, conducted outside developing countries, did not report/measure cognitive outcomes, or targeted children aged >8 years (Fig. 1). The new literature search, therefore, included 23 studies eligible for this meta-analysis. Adding this to the original DFID review, there were a total of 48 studies included, covering the period from 1992 to 2016.

The 48 included studies were conducted in 20 developing countries: Bangladesh, Chile, China, Colombia, Gambia, Ghana, Guatemala, India, Indonesia, Jamaica, Malawi, Mexico, Nepal, Pakistan, Peru, South Africa, Tanzania, Thailand, Viet Nam, and Zambia. All studies except three^S16, S27, S45^ were randomised controlled trials. Five studies targeted at-risk groups, including full-term low-birth-weight,^S37^ low weight-for-age,^S41^ low height-for-age,^S17, S18, S24^ and iron deficiency anaemia.^S10, S15^ These conditions were included in this meta-analysis because of their prevalence in developing countries.

We identified 67 interventions from the 48 studies: 44 on childhood supplementation, 19 on antenatal nutritional supplements, and 4 on both antenatal and childhood supplements. A total of 16944 and 12870 children were in the intervention and control arms respectively (See Appendix Table 1 for detailed information on the studies and interventions).

**Table 1:** Intervention characteristics and cognitive benefits.

	n	effect size	95% CI lower bound	95% CI upper bound	p-value for benefit^a^	p-value for comparison^b^
Childhood supplementation						
Quantity of nutrients						
Single nutrient	15	0.02	−0.05	0.09	0.50	Reference
2–4 nutrients	17	0.05	−0.03	0.12	0.26	0.65
≥5 nutrients	16	0.15	0.08	0.22	< 0.0001	0.005
Children’s age at start						
0–6 months	6	0.11	−0.04	0.26	0.16	Reference
>6– < 18 months	27	0.09	0.02	0.15	0.009	0.78
≥18 months	15	0.04	−0.06	0.16	0.37	0.55
Duration of supplementation						
<6 months	7	0.10	−0.03	0.24	0.12	Reference
≥6 months	41	0.08	0.02	0.13	0.007	0.69
Follow-up time ^c^						
<5 years	40	0.09	0.03	0.15	0.002	Reference
≥5 years	6	0.06	−0.07	0.18	0.36	0.63
Antenatal supplementation						
Quantity of nutrients						
Single nutrient	8	−0.03	−0.10	0.03	0.36	Reference
2–4 nutrients	4	0.03	−0.07	0.14	0.57	0.32
≥5 nutrients	11	0.05	−0.002	0.09	0.06	0.0496
Gestation age at start ^d^						
≤12 weeks	4	0.15	0.03	0.28	0.02	Reference
13–16 weeks	9	0.02	−0.03	0.07	0.38	0.06
≥17 weeks	7	−0.01	−0.08	0.07	0.84	0.03
Follow-up time ^e^						
<5 years	19	0.005	−0.04	0.05	0.94	Reference
≥5 years	5	0.10	0.02	0.19	0.02	0.04

^a^p-value for testing whether the intervention effects were significant.

^b^p-value for comparing the intervention benefits between groups in meta-regressions.

^c^Two childhood supplementation were excluded from this analysis, because they used age to acquire developmental milestone as an outcome measure.

^d^Three antenatal supplementation were excluded from this analysis, because they did not clearly specify the gestation age of pregnant women at the start of interventions.

^e^One intervention was counted twice in this analysis (even though the cluster effects were adjusted through random intercepts), because it had two follow-ups with the first at 18 months and the second at 5 years.

### Cognitive benefits of nutritional supplementation

**Figure 2:**
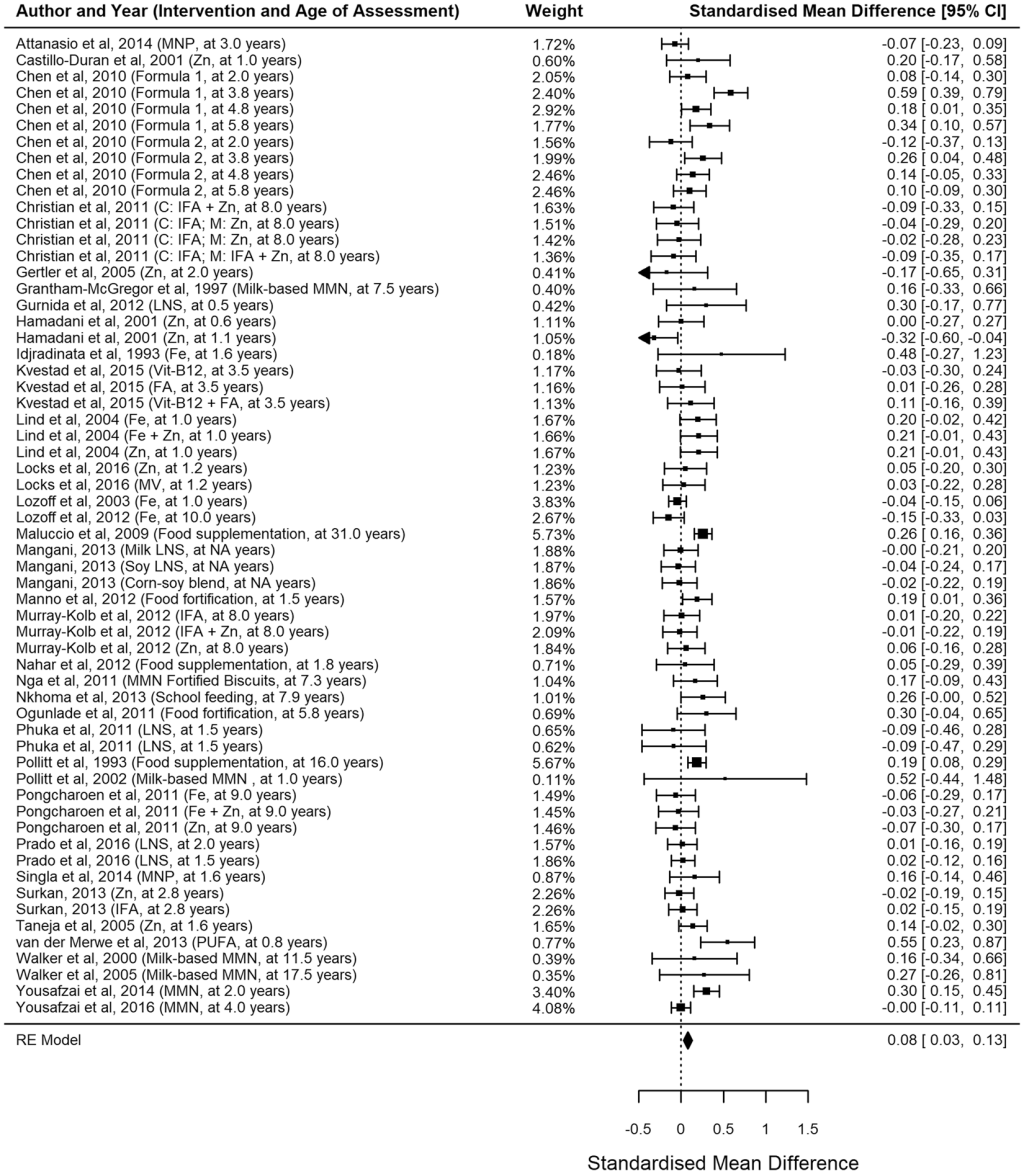
Forest plots of childhood supplementation.

**Figure 3:**
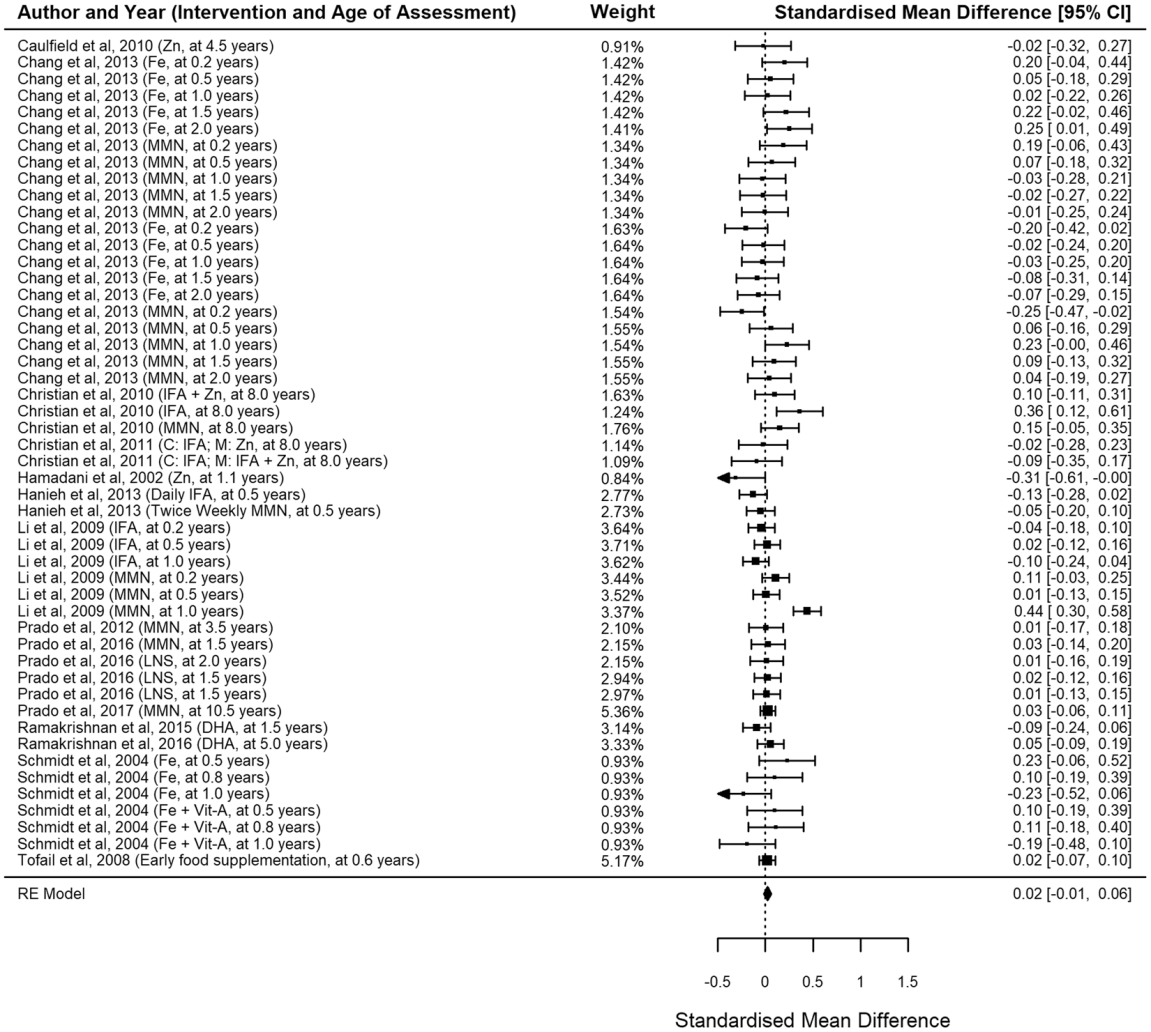
Forest plots of antenatal supplementation.

There were 48 childhood and 23 antenatal nutritional interventions (including the three with both antenatal and childhood supplements). We found a significant pooled effect size of 0.08 (n = 48, 95% CI 0.03–0.13, p = 0.002; Fig. 2) with moderate heterogeneity (I^2^ 53.97, p < 0.0001) in childhood interventions, and a pooled effect size of 0.02 (n = 23, 95% CI −0.01 to 0.06, p = 0.20; Fig. 3) with moderate heterogeneity (I^2^ 47.45, p < 0.0001) in antenatal interventions. Excluding the studies with no random treatment allocations, the pooled effect size for childhood supplementation was slightly lower but was still significant (n = 44, d 0.05, 95% CI 0.004–0.10, p = 0.03).

Childhood nutritional supplements included iron (25 interventions), zinc (23), folic acid (18), lipid/fat (13), calcium (14), vitamin A (14), vitamin B_2_ (13), protein (11), and vitamins B_1_, B_3_, B_12_ (9). Antenatal nutritional supplements given to mothers during pregnancy included zinc (15), iron (10), vitamin A (10), vitamins B_1_, B_2_, B_3_, B_12_ (10), vitamin C (9), and iodine and selenium (8).

### Moderation analysis

The number of nutrients provided was significantly associated with the supplementation benefits (Table 1). Childhood supplementation which included five or more different nutrients produced a significantly larger effect size of 0.15 (n = 16, 95% CI 0.08–0.22, p < 0.0001) compared with 0.02 (n = 15, 95% CI −0.05 to 0.09, p = 0.50) from single-nutrient supplementation (p = 0.005). A similar pattern was observed in antenatal supplementation with marginal statistical significance (p = 0.0496).

Timing of nutritional supplements was also associated with the cognitive benefits of supplementation (Table 1). Antenatal supplementation appeared to have the strongest benefit when started in the first trimester (n = 4, d 0.15, 95% CI 0.03–0.28, p = 0.02); supplementation started later did not yield any significant benefits. Similarly, supplementation on children aged 6–18 months had significant benefit (n = 27, d 0.09, 95%CI 0.02–0.15, p = 0.009) whereas those of older children did not (n = 15, d 0.04, 95% CI −0.06 to 0.16, p = 0.37).

Duration of follow-up (i.e. the time between intervention completion and outcome assessment) was significantly associated with the benefits of antenatal supplementation. Interventions with ≥5 years of follow-up had significantly stronger benefits (n = 5, d 0.10, 95% CI 0.02–0.19, p = 0.02) than the others (n = 19, d 0.005, 95% CI −0.04 to 0.05, p = 0.94). Such phenomenon was not observed in childhood supplementation.

In childhood interventions, several nutrient types were associated with cognitive benefits: iron (n = 25, d 0.09, 95% CI 0.03–0.15, p = 0.01; Table 2), zinc (n = 23, d 0.09, 95% CI 0.02–0.15, p = 0.01), calcium (n = 14, d 0.14, 95% CI 0.07–0.21, p = 0.0002), vitamin B_2_ (n = 13, d 0.11, 95% CI 0.03–0.19, p = 0.01), and protein (n = 11, d 0.13, 95% CI 0.03–0.22, p = 0.01). On the other hand, antenatal supplementation was found effective when they had iron (n = 10, d 0.05, 95% CI 0.002–0.10, p = 0.04), vitamins B_1_, B_2_, B_3_, B_12_ (n = 10, d 0.05, 95% CI 0.01–0.10, p = 0.02), and vitamin C (n = 9, d 0.07, 95% CI 0.02–0.12, p = 0.004).

**Table 2:** Nutritional content and cognitive benefits.

	n	effect size	95% CI lower bound	95% CI upper bound	p-value
Childhood supplementation					
Iron	25	0.09	0.03	0.15	0.01
Zinc	23	0.09	0.02	0.15	0.01
Calcium	14	0.14	0.07	0.21	0.0002
Iodine and Selenium	10	0.06	−0.05	0.17	0.27
Vitamin A	14	0.07	−0.02	0.16	0.15
Vitamin B_1_, B_3_, B_12_	9	0.08	−0.04	0.19	0.20
Vitamin B_2_	13	0.11	0.03	0.19	0.01
Vitamin B_5_	9	0.06	−0.05	0.17	0.28
Vitamin C	8	0.08	−0.03	0.19	0.14
Vitamin D	9	0.06	−0.05	0.17	0.28
Folic Acid	18	0.05	−0.04	0.14	0.27
Lipid/Fat	13	0.07	−0.03	0.18	0.17
Protein	11	0.13	0.03	0.22	0.01
Antenatal supplementation					
Iron	10	0.05	0.002	0.10	0.04
Zinc	15	0.04	−0.004	0.08	0.07
Iodine and Selenium	8	0.05	−0.001	0.10	0.054
Vitamin A	10	0.04	0.00	0.09	0.06
Vitamin B_1_, B_2_, B_3_, B_12_	10	0.05	0.01	0.10	0.02
Vitamin C	9	0.07	0.02	0.12	0.004

Funnel plots and Egger regression tests showed no publication bias in both childhood and antenatal supplementation (z 0.64 and −0.006, p = 0.52 and 1.00; Appendix Figs 2 and 3).

## Discussion

In our meta-analysis of 67 interventions, childhood nutritional supplementation was found to be generally effective in improving cognitive development of children in developing countries. This finding was also supported by the previous preliminary evidence^13^. Even more importantly, we found that timing of supplementation, number of nutrients, and some specific types of nutrients were associated with better effectiveness.

Number of nutrients seemed to be a crucial factor for optimal cognitive development. Supplementation with five or more nutrients had much stronger benefits than those with single nutrient. This could be related to the fact that multiple nutritional deficiency or insufficiency is relatively common in developing countries^17^. Provision of multiple nutrients is more likely to bridge the gap and prepare the optimal foundation for rapid brain development in early childhood.

Childhood supplementation of iron, zinc, calcium, vitamins B_2_ and proteins were found to be particularly effective in improving cognitive outcomes. Although the exact mechanisms are still poorly understood, this could be related to the roles of these nutrients in early brain development^18^. For example, protein plays a critical role in brain growth and advancement in cognitive abilities^6^, and vitamin B_2_ (riboflavin) is required for metabolising fatty acids by brain issues^19, 20^, which are essential for brain development.

Timing of nutritional implementation appeared to be important to cognitive outcomes as well. Supplementation programmes implemented from 6 to 18 months of age had the largest benefit among all age groups. The supplementation of nutrients to young, malnourished children may provide them with necessary resources for rapid brain development during the first year of age. This important finding on timing of intervention should be factored into future programme design^21^.

The supplementation for children aged 18 months and above were found to be ineffective to their cognitive outcomes. Nonetheless, we should be cautious that this group of children were assessed with heterogenous measurements. Some were assessed with cognitive/executive function tests, while some with school readiness/achievement. We should also note that many of these studies only provided single or relatively few nutrients.^S41, S44, S47^ Supplementation provided multi-nutrient food fortification^S42, S43^ or early primary school feeding^S45^ did achieve a small to medium effects (0.17–0.30) for this group of children, even though a study supplementing multi-nutrient powder^S46^ did not have significant effect (−0.07). This finding is consistent with the current understanding of brain development in the early ages. While our brain is developed most rapidly in the first year of life, the neural connections for higher cognitive circuits are still actively formed throughout the earlier years until middle childhood^22^. To understand what kind of supplementation would benefit these older children, future trials may consider multi-nutrient food fortification and feeding programmes.

Consistent with previous studies on antenatal supplementation^23^, we found minimal effects of antenatal nutrition supplementation on children’s cognitive development. However, pre-specified subgroup analysis showed that nutritional supplementation in first trimester of pregnancy actually benefited children’s cognitive development. This finding suggests that some of the previous trials may have missed the critical window for intervention in early pregnancy.

It is also worth noting that this meta-analysis may have underestimated the true potential of antenatal supplementation, as most studies included had only short-term assessments but the true benefits of supplementation may only become apparent after a longer period. In this meta-analysis, the long-term cognitive functions assessed five years after antenatal supplementation showed a much larger effect size, more than twice of those with short-term assessment. This evidence echoes with the conclusion of Copenhagen Consensus that nutritional interventions had substantial benefits to children’s cognitive development and long-term productivity, which could yield a high rate of economic return^24^.

The following limitations should be noted. First, three of the studies in the meta-analysis were not randomised controlled trials. There may have potential bias related to underlying confounders. However, it should not substantially affect our results and conclusion given their small proportion in the whole meta-analysis. Second, the exact timing of cognitive assessments varied among studies which may make comparison of outcomes difficult. However, most studies assessed children’s cognitive performance soon after the programme and within one year of the supplementation. Meta-regression analysis was also conducted to address the potential heterogeneity due to the timing of assessment. Third, details on the participants’ baseline cognitive abilities and compliance to nutritional supplementation were not available in most publications. Future trials should pay more attention in reporting this important information. Fourth, the analysis of dosage cannot be reliably conducted because some studies did not report the dosage used and some had time-varying dosage. Fifth, some of the moderation analysis were possibly underpowered (e.g. quantity of nutrients for antenatal supplementation), so the statistical insignificance did not necessarily prove a null effect. Sixth, keywords such as ‘developing country’ and ‘low- and middle-income country’ were used to limit the number of search results, which could have excluded some relevant studies. Findings of some specific nutrients, such as iodine, may not be complete. Last but not least, the study focused on early interventions and excluded most of the school feeding programmes. The impact of these school feeding programmes need to be studied in a future meta-analysis using a broader age criterion.

In conclusion, this meta-analysis provides robust evidence on the benefits of nutritional supplements on children’s cognitive outcomes in developing countries. Future supplementation should aim to provide multiple nutrients to younger children and pregnancy women at the first trimester. To identify the optimal supplementation, future studies should include both short- and long-term assessments on the cognitive performance of children and consider the potential interactive effects of different nutrients.

## Methods

The current study was an extension of a literature review commissioned by the Department for International Development, UK (DFID) in 2013, which included searching of studies published from 1 January 1992 to 31 December 2012. Detailed methodology of that commissioned study was published elsewhere^25^, which focused on the cognitive benefits of early childhood development programmes. The present study further reviewed the original studies and extracted additional information that were not available or reported previously^4^. This study also searched for nutritional supplementation interventions published from 1 January 2013 to 31 December 2016, conducted original analysis, and reported new findings, including the separation of antenatal from childhood nutritional supplementation and the investigation of supplementation programme characteristics in detail.

### Study identification strategy and selection criteria

The systematic review and meta-analysis was conducted in accordance with PRISMA guidelines. The systematic literature search examined quantitative evidence on the benefits of nutritional supplementation implemented from pregnancy to 8 years of age on cognitive development in children in developing countries.

The identification of studies followed the Evidence for Policy and Practice Information and Co-ordinating Centre’s guidelines^26^ using four approaches: searching electronic databases, manually searching key journals, searching specialist websites, and asking experts in the field. Nine electronic databases were searched by specific keywords in the original DFID study: Academic Search Elite/EBSCOhost, Cochrane Reviews, Google Scholar, JSTOR, ProQuest, PubMed, PsycINFO, The University of Hong Kong Libraries Catalogue, and Web of Science. Keywords reflecting early childhood interventions included *early childhood programme*, *early intervention*, and *early nutritional supplementation*. Keywords reflecting cognitive development included *school readiness, cognitive development, academic achievement*, and *intelligence* (see Appendix for a list of keywords). Publications cited in the reference lists of selected papers and reviews were manually searched. Specialist websites: UNICEF Evaluation Database, UNESCO, World Bank, Brookings Institute, Save the Children, Bernard van Leer, National Institute of Early Education Research (NIEER), Consultative Group on Early Childhood Care and Development, Young Lives, Pratham, International Initiative for Impact Evaluation (3ie), Open Society Institute, and Plan International were searched for conference proceedings, research reports, and policy papers. Since there was a significant overlapping in the nine databases, we decided to search for four key databases in the search of papers published from 2013 to 2016: Academic Search Elite/EBSCOhost, Google Scholar, PsyINFO, and PubMed. A validation search for papers published in 2012 have found that the two searching strategies yielded identical results. After searching the specific keywords in the databases, the titles were firstly screened, which was then followed by abstract screening, and subsequently full-text screening. Six coders worked on these procedures with more than 20% of the screening results randomly cross-checked to ensure inter-rater reliability.

A complete list of inclusion and exclusion criteria can be found in the Appendix. Briefly, studies were included if they were interventions conducted in developing countries (listed by the World Bank as low or middle income countries) and included quantitative evaluations of children’s cognitive development, including global cognitive/mental development (e.g. Mental Development Index of Bayley Scales of Infant Development II), intelligent quotients (e.g. Wechsler Intelligence Scale for Children), language development (e.g. Language Scale in Bayley Scales of Infant and Toddler Development III), executive function (e.g. Stroop Test), academic performance (e.g. standardised language or mathematics test in school), and time to developmental milestone acquisition (e.g. age at which the child can say simple words). Studies targeting special populations, such as Down’s syndrome, cerebral palsy, autism, and any disability, or studies with deficiencies in sampling, data collection, or data analysis after quality assessment were excluded (See Appendix for quality assessment criteria). The current meta-analysis only included those early childhood interventions (either randomised controlled trials, clustered randomised trials, or quasi-experiments) with pure nutritional supplementation, i.e. the difference between intervention and control arms was only nutrient or food supplementation. Interventions were excluded if the effect of supplementation was confounded with other non-supplementation components (e.g. direct stimulation or parenting education).

### Data extraction

Intervention was regarded as the unit of analysis. Effect sizes were calculated by comparing differences in cognitive outcomes between intervention and control groups. Multiple relevant outcomes (e.g. Stroop Test and Go/No-Go Test) at a single time point were averaged. Assessment at multiple time points (except for interim analyses before the completion of intervention, which were excluded) were included separately. Unadjusted effect sizes were extracted from randomised controlled trials and adjusted effect sizes were preferably extracted from other studies. The following intervention characteristics were considered as potential effect moderators: type and quantity of nutrients, children’s age (gestational age for antenatal supplementation) at the start of supplementation, and duration of supplementation. Age of cognitive outcome evaluation was also extracted in this study to test whether the effect of nutritional supplementation may change over time. Details on each of the interventions were coded by six coders. Over half of the codes were then randomly selected for verification. At the end of this process, the coding was independently reviewed by all team members.

### Statistical analysis

We used the standardised mean difference (Cohen’s *d*) with Hedges and Olkin’s bias correction as the effect size^27^. Because of the heterogeneity in cognitive outcome measurements, random-effects meta-analysis models were used with the inverse of effect size precision (variance) as the weighting. Random-effects meta-regression models with restricted maximum likelihood (REML) estimators were used to test the statistical significance of potential moderators^28^. One meta-regression was constructed for each moderator variable. To address for intra-class correlation due to multiple follow-up time points and multiple publications of a single study, both the study identification number and the intervention identification number were included as random intercepts. Childhood and antenatal interventions were analysed separately. Potential moderators except nutritional content were pre-specified by the authors based on clinical experience and the extant literature. Nutritional content variables were extracted from each intervention and grouped accordingly. Heterogeneity of intervention effect sizes was examined using Cochran’s Q tests and I^2^ statistics. Publication bias was separately assessed for childhood and antenatal supplementation using funnel plots and Egger regression tests.

## Electronic supplementary material

Supplementary information
