# Supplementary material for: Impact of nutritional supplements on cognitive development of children in developing countries: A meta-analysis
Source: Sci Rep. 2017 Sep 6;7:10611. doi: 10.1038/s41598-017-11023-4 (PMC5587553; doi:10.1038/s41598-017-11023-4)
Supplement: Supplementary file 1 — Supplementary information [file 41598_2017_11023_MOESM1_ESM.pdf]

Impact of nutritional supplements on cognitive development of children in developing countries: A meta-analysis

Patrick Ip[1], Frederick Ka Wing Ho[1], Nirmala Rao[2], Jin Sun[3], Mary Eming Young[4], Chun Bong Chow[1], Winnie Tso[1], Kam Lun Hon[5]

Affiliations: [1]Department of Paediatrics and Adolescent Medicine; [2]Faculty of Education, The University of Hong Kong; [3]Department of Early Childhood Education, Hong Kong Institute of Education, Hong Kong; [4]Center on the Developing Child, Harvard University, Cambridge, Massachusetts; [5]Department of Paediatrics, The Chinese University of Hong Kong

Online Appendix

List of contents | Page

List of contents	Page
Keywords for electronic database search in DFID Rigorous Literature Review	2
Detailed inclusion and exclusion criteria in DFID Rigorous Literature Review	4
Quality Assessment Coding	5
Table 1. Studies and interventions included	6
Figure 1. Literature search in original DFID review for papers published 1992–2012	9
Figure 2. Funnel plot for childhood supplementations	10
Figure 3. Funnel plot for antenatal supplementations	11
References	12

Keywords for electronic database search in DFID Rigorous Literature Review

The following keywords were used for the electronic database search in the original rigorous literature review for articles published in 1992–2013, from which we extracted studies on nutritional supplementation for our meta-analysis. The keywords for searching used the intersection (AND operator) of keyword groups:

1. Union (OR operator) of these keywords for developing countries
    1.1. developing countr*
    1.2. low-income countr*
    1.3. mid-income countr*
2. Union of these keywords for children
    2.1. child*
    2.2. infant*
3. Union of these keywords for cognitive development
    3.1. academic achievement
    3.2. attention
    3.3. basic concept*
    3.4. brain develop*
    3.5. child develop*
    3.6. cognitive develop*
    3.7. communication skills
    3.8. development quotient
    3.9. DQ
    3.10. executive function*
    3.11. intelligen*
    3.12. IQ
    3.13. language develop*
    3.14. learning outcome*
    3.15. literacy
    3.16. mathematics achievement
    3.17. neural develop*
    3.18. problem solving
    3.19. school readiness
    3.20. thinking

3.21. vocabulary

4. Union of these keywords for interventions
    4.1. Intervention
    4.2. Program*
    4.3. Experiment
    4.4. Trial
    4.5. Education
    4.6. Stimulation
    4.7. Supplementation
    4.8. Food fortification
    4.9. Feeding Programme

Detailed inclusion and exclusion criteria in DFID Rigorous Literature Review

The inclusion and exclusion criteria of the *original rigorous literature review*, from which we extracted studies on nutritional supplementation for our meta-analysis.

1. The interventions must have begun during early childhood; that is, before the children were 8 years of age.

2. The interventions must contain at least one of the following components: (a) parent-focused education and support; (b) child-focused education and stimulation; (c) nutrition and health; and (d) income supplementation including cash transfers.

3. The interventions could be home-, centre-, and/or community-based. Centre-based approaches involved several kinds of institutions offering early years provision such as preschools, childcare centres, crèches, playgroups, day care nurseries, and nursery schools, which served as alternative physical and social environments for care, development, and education.

4. The interventions must have explicitly documented cognitive and/or schooling outcomes.

5. The evidence assessed linkages between participation in the interventions and cognitive outcomes.

6. The studies were published after 1st January 1992 and before 31st December 2012.

7. The studies provided information from a primary study which was not a literature review.

8. Research methods, statistical analyses, and findings were sufficiently detailed to provide a basis for judgment about the robustness of the conclusions; that is, the research procedures and characteristics of the sample were specified in detail, so that the validity of the results could be evaluated.

9. Comparisons (concurrent between groups or before-and-after within groups) among groups of people exposed to the intervention and those who were not exposed or less exposed to the intervention were available.

10. Studies involving special populations such as Down's syndrome, cerebral palsy, autism or any specific form of disability (sensory, physical, intellectual, or psychological), and extreme malnutrition were not included.

Quality Assessment Coding

Rigor of study: Level of rigor in study design

Code	Meaning
1	Other, lowest level of rigor
2	Single group before and after
3	Retrospective controlled/secondary data analysis/construction of comparison groups
4	Other prospective quasi-experimental design
5	Quasi-experimental design with the use of an econometric model
6	Randomized controlled trial

Soundness of study: Level of validity and reliability of the study

Code	Meaning
1	Low (major and or numerous deficiencies in sampling, data collection, or data analysis)
2	Medium (some deficiencies in sampling, data collection, or data analysis, but the methods and interpretations were generally valid and reliable)
3	High (demonstrates adherence to appropriate sampling and data collection methods and reliable data analysis)

Overall quality of study: Code of Rigor + Code of Soundness

Studies with overall quality <6 were excluded from this study.

Table 1. Studies and interventions included

Ref.	Country / region	Year of publication	Study quality	Test	Intervention arm	Type of intervention	$N_{intervention}$	$N_{control}$	$d_{unbiased}$
					**Studies with at least one combined antenatal and childhood supplementation**				
S1	Nepal	2011	9	EF tests, UNIT	Iron, Folic acid, Zinc	Childhood (≥18m)	212	101	-0.09
					Iron, Folic acid	Childhood (≥18m)	163		-0.04
					Antenatal: Zinc; Childhood: Iron, Folic acid	Antenatal + Childhood (≥18m)	137		-0.02
					Antenatal: Zinc; Childhood: Iron, Folic acid, Zinc	Antenatal + Childhood (≥18m)	122		-0.09
S2	Malawi	2016	9	EF test	Multi-nutrients (Zinc, Iron, Selenium, and Multivitamins)	Antenatal	251	251	0.03
					Lipid-based nutrients (Protein, Fat, Zinc, Iron, Selenium, and Multivitamins)	Antenatal + Childhood (<18m)	253		0.02
S3	Ghana	2016	8	Vocabulary checklist, EF tests	Lipid-based nutrients	Antenatal + Childhood (<18m)	387	389	0.02
					Multi-nutrients	Antenatal	397		0.01
					**Studies with antenatal supplementation**				
S4	Bangladesh	2002	9	BSID II	Zinc	Antenatal	83	85	-0.31
S5	Bangladesh	2008	9	One-step means-end problem-solving test	Food supplementation	Antenatal	1058	1058	0.02
S6	China	2009	9	BSID	Iron	Antenatal	392	422	-0.04
					Multi-nutrients (including Iron and Multivitamins)	Antenatal	350		0.11
S7	Peru	2010	9	Self-developed tests on language and pre-arithmetic, WPPSI	Zinc	Antenatal	85	78	-0.02
S8	Nepal	2010	9	EF tests, UNIT	Iron, Folic Acid, Zinc	Antenatal	178	177	0.10
					Iron, Folic Acid	Antenatal	103		0.36
					Multi-nutrients (Folic acid, Iron, and Multivitamins)	Antenatal	218		0.15
S9	Indonesia	2012	9	EF tests	Multi-nutrient (Zinc, Copper, Iodine, Selenium, and Multivitamins)	Antenatal	246	241	0.00
S10	China	2013	9	BSID II	Iron [Sample 1]	Antenatal	165	280	0.21
					Multi-nutrients (Zinc, Iron, Copper, Iodine, Selenium, Multivitamins) [Sample 1]	Antenatal	165	269	0.19
					Iron [Sample 2]	Antenatal	148	316	-0.20
					Multi-nutrients (Zinc, Iron, Copper, Iodine, Selenium, Multivitamins) [Sample 2]	Antenatal	148	298	-0.25
S11	Viet Nam	2013	8	BSID III	Iron Folic Acid daily	Antenatal	350	363	-0.13
					Multi-nutrients twice weekly (Zinc, Iodine, Copper, Selenium, Multivitamins)	Antenatal	335		-0.05
S12	Mexico	2015	9	BSID III	DHA	Antenatal	365	365	-0.09

Ref.	Country / region	Year of publication	Study quality	Test	Intervention arm	Type of intervention	$N_{intervention}$	$N_{control}$	$d_{unbiased}$
S13	Mexico	2016	9	McCarthy Scales of Children's Abilities, Conner's' Kiddie Continuous Performance Test	DHA	Antenatal	401	396	0.05
S14	Indonesia	2017[b]	9	Self-developed tests on general intelligence, EF, educational achievement	Multi-nutrients (Zinc, Copper, Iodine, Selenium, Multivitamins)	Antenatal	1187	1118	0.03
**Studies with childhood supplementation (started at <18 months)**									
S15	Indonesia	1993	9	BSID	Iron	Childhood (<18m)	24	23	0.48
S16	Guatemala	1993	6	Achievement tests, RPM	Protein, Fat, Carbohydrate, Calcium, Phosphorous, Iron	Childhood (<18m)	758	652	0.19
S17	Jamaica	1997	9	EF tests, PPVT, RPM, WRAT, SBIT	Multi-nutrient (Milk-based formula with protein)	Childhood (<18m)	31	32	0.16
S18	Jamaica	2000	9	EF tests, PPVT, RPM, WISC-R	Multi-nutrient (Milk-based formula with protein)	Childhood (<18m)	30	31	0.16
S19	Bangladesh	2001	9	BSID	Zinc	Childhood (<18m)	103	109	0.00
S20	Chile	2001	9	BSID II	Zinc	Childhood (<18m)	57	52	0.20
S21	Indonesia	2002	8	BSID II	Milk-based micronutrient	Childhood (<18m)	38	37	0.52
S22	Chile	2003	9	BSID	Iron	Childhood (<18m)	1082	531	-0.04
S23	Indonesia	2004	9	BSID	Iron	Childhood (<18m)	163	164	0.20
					Iron, Zinc	Childhood (<18m)	161		0.21
					Zinc	Childhood (<18m)	162		0.21
S24	Jamaica	2005	9	EF tests, PPVT, RPM, WRAT, WAIS	Multi-nutrient (Milk-based formula with protein)	Childhood (<18m)	28	27	0.27
S25	India	2005	8	BSID II	Zinc	Childhood (<18m)	283	288	0.14
S26	Guatemala	2009	9	Non-verbal cognitive test, Reading test	Protein, Fat, Carbohydrate, Calcium, Phosphorous, Iron	Childhood (<18m)	736	735	0.26
S27	China	2010	7	Development diagnostic scale, WPPSI	Multi-nutrient (Folic acid, Iron, Zinc, Multivitamins)	Childhood (<18m)	232	127	0.08
				Development diagnostic scale, WPPSI	Protein	Childhood (<18m)	116		-0.12
S28	Malawi	2011	9	GMDS	Lipid-based nutrient supplement (50g/day)	Childhood (<18m)	56	56	-0.09
					Lipid-based nutrient supplement (25g/day)	Childhood (<18m)	51		-0.09
S29	Thailand	2011	9	School performance, WISC-III, RCPM	Iron	Childhood (<18m)	147	139	-0.06
					Iron, Zinc	Childhood (<18m)	135		-0.03
					Zinc	Childhood (<18m)	139		-0.07
S30	Bangladesh	2012	8	BSID II	Food supplementation	Childhood (<18m)	77	59	0.05
S31	Chile	2012	9	WISC, KABC, WRAT	Iron	Childhood (<18m)	244	229	-0.15
S32	Indonesia	2012	8	GMDS	Lipid-based nutrient supplement	Childhood (<18m)	35	35	0.30
S33	Zambia	2012	9	BSID II	Food fortification	Childhood (<18m)	246	256	0.19

Ref.	Country / region	Year of publication	Study quality	Test	Intervention arm	Type of intervention	$N_{intervention}$	$N_{control}$	$d_{unbiased}$
S34	Gambia	2013 [a]	9	Toddler attention assessment, 2-step Infant Planning Test	Fish oil (DHA and EPA/d)	Childhood (<18m)	87	85	0.55
S35	Malawi	2013	8	Age to achieve developmental milestone	Milk lipid-based nutrients	Childhood (<18m)	191	185	0.00
					Soy milk lipid-based nutrients	Childhood (<18m)	188		-0.04
					Corn-soy blend	Childhood (<18m)	183		-0.02
S36	Nepal	2013	9	Adapted Child Development Inventory	Zinc	Childhood (<18m)	276	268	-0.02
					Iron, Folic Acid	Childhood (<18m)	268	276	0.02
S37	Bangladesh	2014	8	BSID III	Multi-nutrient powder	Childhood (<18m)	96	82	0.16
S38	Pakistan	2014	9	BSID III	Multi-nutrient powder (Iron, Folic Acid, Vitamin A and C)	Childhood (<18m)	335	348	0.30
S39	Pakistan	2016	9	BSID III	Multi-nutrient powder (Iron, Folic Acid, Vitamin A and C)	Childhood (<18m)	589	648	0.00
S40	Tanzania	2016	9	BSID III	Zinc	Childhood (<18m)	121	126	0.05
					Folic Acid, Multi-vitamin	Childhood (<18m)	119	128	0.03

Studies with childhood supplementation (started at ≥18 months)

Ref.	Country / region	Year of publication	Study quality	Test	Intervention arm	Type of intervention	$N_{intervention}$	$N_{control}$	$d_{unbiased}$
S41	Jamaica	2005	9	GMDS	Zinc	Childhood (≥18m)	30	38	-0.17
S42	South Africa	2011	8	KABC-II	Fortified breakfast maize-porridge	Childhood (≥18m)	63	68	0.30
S43	Viet Nam	2011	9	EF tests, RCM	Multi-nutrients (Iron, Zinc, Iodine, and Multivitamins)	Childhood (≥18m)	114	119	0.17
S44	Nepal	2012	9	EF tests	Iron, Folic acid	Childhood (≥18m)	169	176	0.00
					Iron, Folic acid, Zinc	Childhood (≥18m)	199		-0.01
					Zinc	Childhood (≥18m)	144		0.06
S45	Malawi	2013	6	Cambridge Neurological Test Automated Battery	School feeding programme	Childhood (≥18m)	114	111	0.26
S46	Colombia	2014	9	BSID III	Multi-nutrient powder (Iron, Folic Acid, Vitamin A and C)	Childhood (≥18m)	308	318	-0.07
S47	India	2015	8	ASQ 3	Vitamin B12	Childhood (≥18m)	109	105	-0.03
					Folic Acid	Childhood (≥18m)	107		0.01
					Vitamin B12, Folic Acid	Childhood (≥18m)	101		0.11

[a] The study was published in print in 2013 but was available online in 2012.
[b] The study was published in print in 2017 but was available online in 2016.
Abbreviation for cognitive assessments: ASQ: Ages and Stages Questionnaire; BSID: Bayley Scales of Infant Development; EF tests: executive function tests, such as Stroop Test, Go/no-go Test; GMDS: Griffiths Mental Development Scale; KABC: Kaufman Assessment Battery for Children; PPVT: Peabody Picture Vocabulary Test; RCPM: Raven's Coloured Progressive Matrices; RPM: Raven's Progressive Matrices; SBIT: Stanford-Binet Intelligence Test; UNIT: Universal Nonverbal Intelligence Test; WAIS: Wechsler Adult Intelligence Scale; WISC: Wechsler Intelligence Scale for Children; WPPSI: Wechsler Preschool and Primary Scale of Intelligence; WRAT: Wide Range Achievement Test

Figure 1. Literature search in original DFID review for papers published 1992–2012

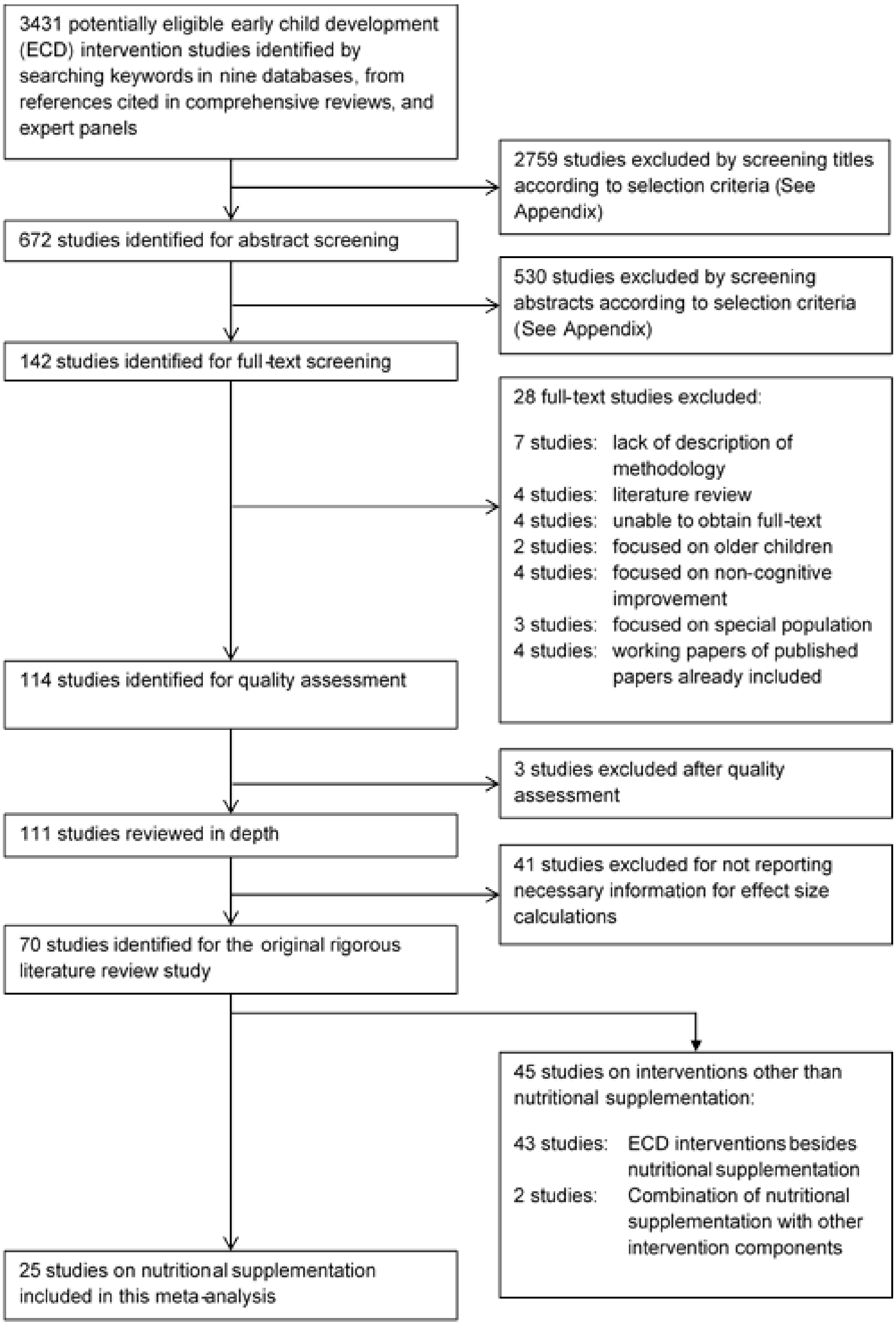

Altogether 3431 candidate studies were identified using the four search strategies. After screening the abstracts and full articles, 111 studies fulfilled the criteria and with good quality received in-depth review and 41 studies without information for effect size calculation were excluded. Among the remaining 70 studies, 43 without nutritional supplementations and two with combination of educational and nutritional interventions were further excluded. As a result, 25 studies published from 1993 to 2012 were included in the final meta-analysis (see Figure 1 for the identification procedure).

Figure 2. Funnel plot for childhood supplementations

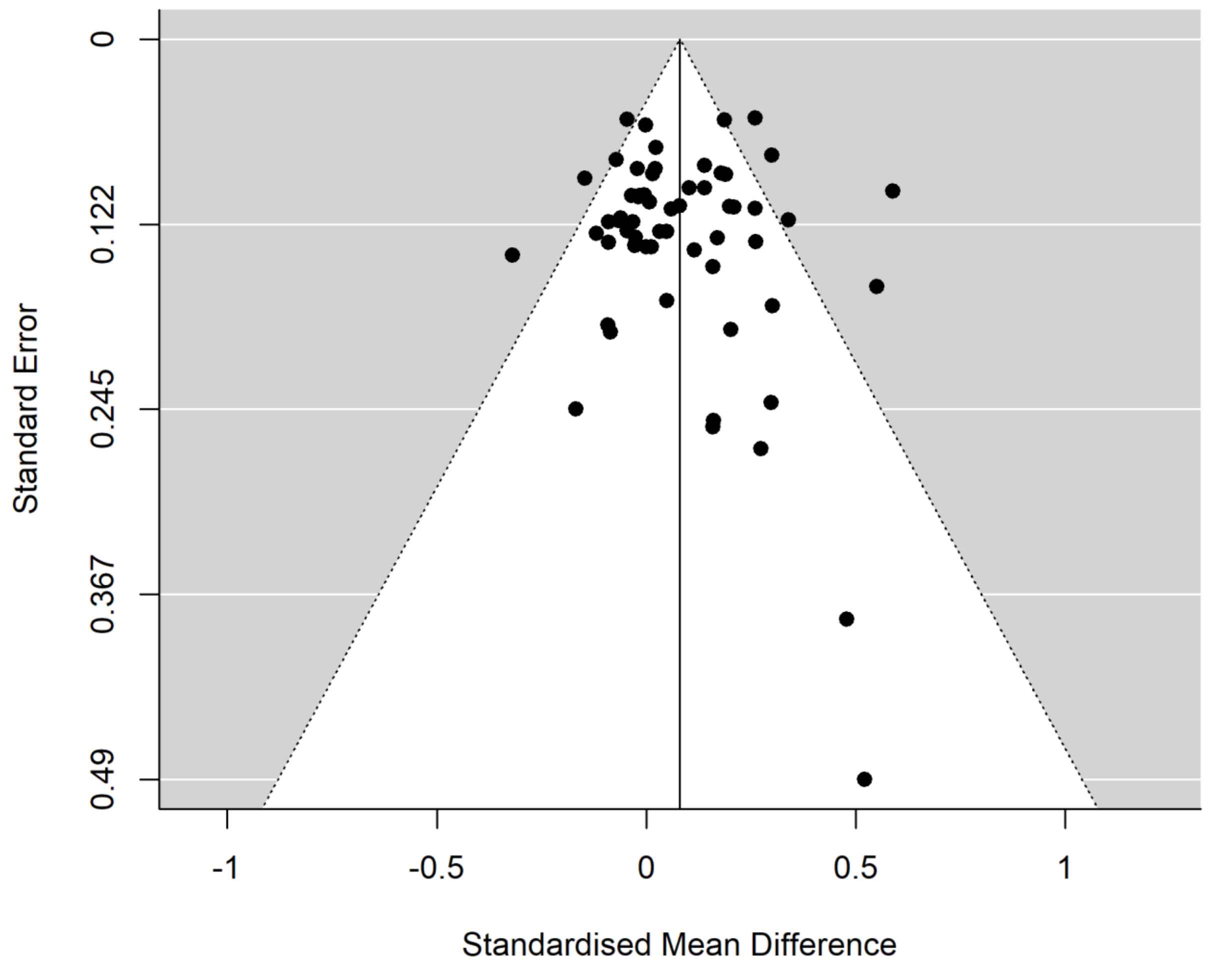

Egger's regression test reported no significant asymmetry in the funnel plot (z 0.64, p=0.52).

Figure 3. Funnel plot for antenatal supplementations

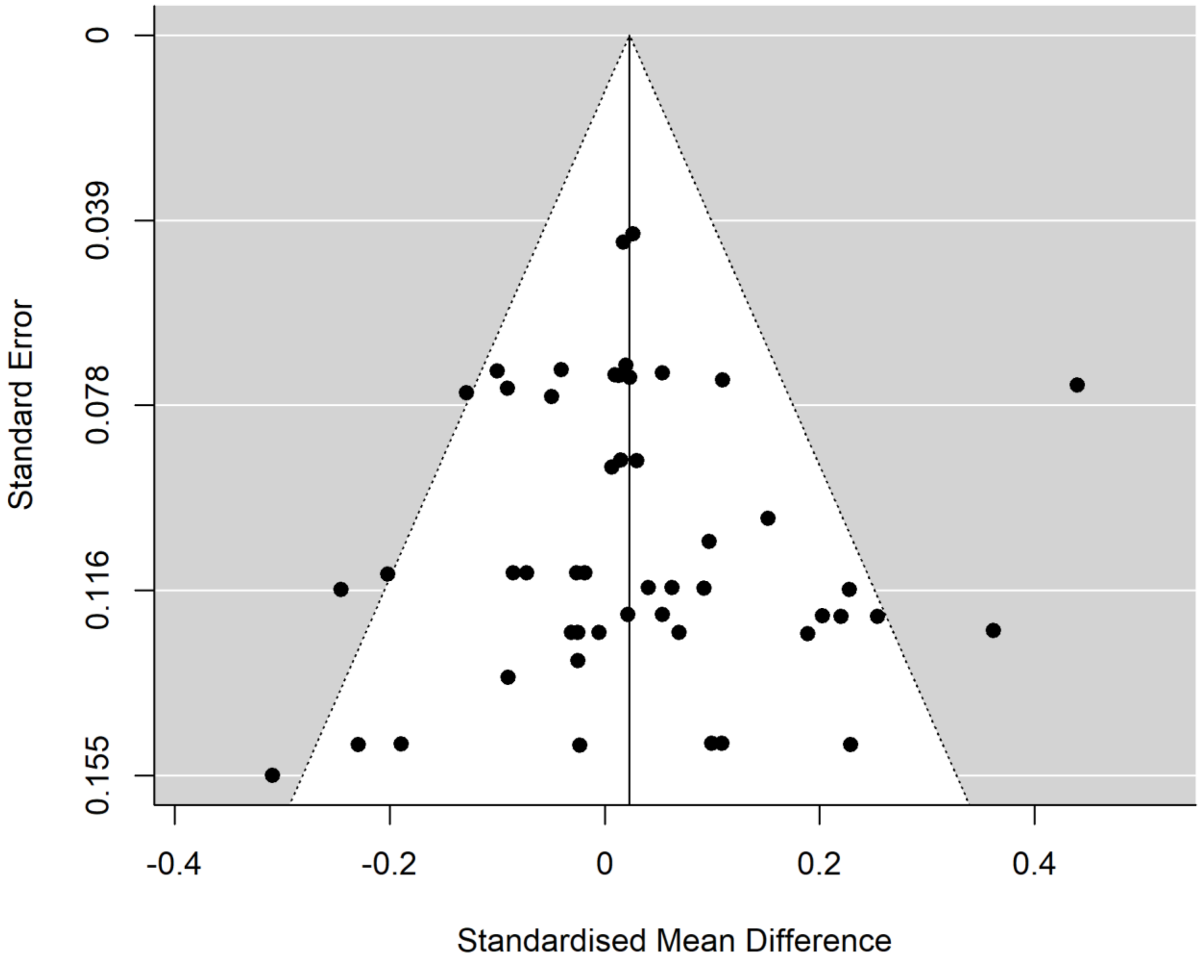

Egger's regression test reported no significant asymmetry in the funnel plot (z -0.006, p=1.00).

References

S1. Christian P, Morgan ME, Murray-Kolb L, et al. Preschool Iron-Folic Acid and Zinc Supplementation in Children Exposed to Iron-Folic Acid in Utero Confers No Added Cognitive Benefit in Early School-Age. *J Nutr* 2011; **141**(11): 2042-8.

S2. Prado EL, Maleta K, Ashorn P, et al. Effects of maternal and child lipid-based nutrient supplements on infant development: a randomized trial in Malawi. *Am J Clin Nutr* 2016; **103**(3): 784-93.

S3. Prado EL, Adu-Afarwuah S, Lartey A, et al. Effects of pre-and post-natal lipid-based nutrient supplements on infant development in a randomized trial in Ghana. *Early Hum Dev* 2016; **99**: 43-51.

S4. Hamadani JD, Fuchs GJ, Osendarp SJM, Huda SN, Grantham-McGregor SM. Zinc supplementation during pregnancy and effects on mental development and behaviour of infants: a follow-up study. *Lancet* 2002; **360**(9329): 290-4.

S5. Tofail F, Persson LÅ, El Arifeen S, et al. Effects of prenatal food and micronutrient supplementation on infant development: a randomized trial from the Maternal and Infant Nutrition Interventions, Matlab (MINIMat) study. *Am J Clin Nutr* 2008; **87**(3): 704-11.

S6. Li Q, Yan H, Zeng L, et al. Effects of Maternal Multimicronutrient Supplementation on the Mental Development of Infants in Rural Western China: Follow-up Evaluation of a Double-Blind, Randomized, Controlled Trial. *Pediatrics* 2009; **123**(4): e685-e92.

S7. Caulfield LE, Putnick DL, Zavaleta N, et al. Maternal gestational zinc supplementation does not influence multiple aspects of child development at 54 mo of age in Peru. *Am J Clin Nutr* 2010; **92**(1): 130-6.

S8. Christian P, Murray-Kolb LE, Khatry SK, et al. Prenatal micronutrient supplementation and intellectual and motor function in early school-aged children in nepal. *JAMA* 2010; **304**(24): 2716-23.

S9. Prado EL, Alcock KJ, Muadz H, Ullman MT, Shankar AH. Maternal Multiple Micronutrient Supplements and Child Cognition: A Randomized Trial in Indonesia. *Pediatrics* 2012; **130**(3): e536-e46.

S10. Chang S, Zeng L, Brouwer ID, Kok FJ, Yan H. Effect of iron deficiency anemia in pregnancy on child mental development in rural China. *Pediatrics* 2013; **131**(3): e755-e63.

S11. Hanieh S, Ha TT, Simpson JA, et al. The effect of intermittent antenatal iron supplementation on maternal and infant outcomes in rural Viet Nam: a cluster randomised trial. *PLoS Med* 2013; **10**(6): e1001470.

S12. Ramakrishnan U, Stinger A, DiGirolamo AM, et al. Prenatal docosahexaenoic acid supplementation and offspring development at 18 months: randomized controlled trial. *PLoS One* 2015; **10**(8): e0120065.

S13. Ramakrishnan U, Gonzalez-Casanova I, Schnaas L, et al. Prenatal supplementation with DHA improves attention at 5 y of age: a randomized controlled trial. *Am J Clin Nutr* 2016; **104**(4): 1075-82.

S14. Prado EL, Sebayang SK, Apriatni M, et al. Maternal multiple micronutrient supplementation and other biomedical and socioenvironmental influences on children's cognition at age 9–12 years in Indonesia: follow-up of the SUMMIT randomised trial. *Lancet Glob Health* 2017; **5**(2): e217-e28.

S15. Idjradinata P, Pollitt E. Reversal of developmental delays in iron-deficient anaemic infants treated with iron. *Lancet* 1993; **341**(8836): 1-4.

S16. Pollitt E, Gorman KS, Engle PL, et al. Early supplementary feeding and cognition: effects over two decades. *Monogr Soc Res Child Dev* 1993: 1-118.

S17. Grantham-McGregor SM, Walker SP, Chang SM, Powell CA. Effects of early childhood supplementation with and without stimulation on later development in stunted Jamaican children. *Am J Clin Nutr* 1997; **66**(2): 247-53.

S18. Walker SP, Grantham-McGregor SM, Powell CA, Chang SM. Effects of growth restriction in early childhood on growth, IQ, and cognition at age 11 to 12 years and the benefits of nutritional supplementation and psychosocial stimulation. *J Pediatr* 2000; **137**(1): 36-41.

S19. Hamadani JD, Fuchs GJ, Osendarp SJ, Khatun F, Huda SN, Grantham-McGregor SM. Randomized controlled trial of the effect of zinc supplementation on the mental development of Bangladeshi infants. *Am J Clin Nutr* 2001; **74**(3): 381-6.

S20. Castillo-Durán C, Perales CG, Hertrampf ED, Marín VB, Rivera FA, Icaza G. Effect of zinc supplementation on development and growth of Chilean infants. *J Pediatr* 2001; **138**(2): 229-35.

S21. Pollitt E, Jahari A, Husaini M, Kariger P, Saco-Pollitt C. Developmental trajectories of poorly nourished toddlers that received a micronutrient supplement with and without energy. *J Nutr* 2002; **132**(9): 2617-25.

S22. Lozoff B, De Andraca I, Castillo M, Smith JB, Walter T, Pino P. Behavioral and Developmental Effects of Preventing Iron-Deficiency Anemia in Healthy Full-Term Infants. *Pediatrics* 2003; **112**(4): 846-54.

S23. Lind T, Lönnerdal B, Stenlund H, et al. A community-based randomized controlled trial of iron and zinc supplementation in Indonesian infants: effects on growth and development. *Am J Clin Nutr* 2004; **80**(3): 729-36.

S24. Walker SP, Chang SM, Powell CA, Grantham-McGregor SM. Effects of early childhood psychosocial stimulation and nutritional supplementation on cognition and education in growth-stunted Jamaican children: prospective cohort study. *Lancet* 2005; **366**(9499): 1804-7.

S25. Taneja S, Bhandari N, Bahl R, Bhan MK. Impact of zinc supplementation on mental and psychomotor scores of children aged 12 to 18 months: A randomized, double-blind trial. *J Pediatr* 2005; **146**(4): 506-11.

S26. Maluccio JA, Hoddinott J, Behrman JR, Martorell R, Quisumbing AR, Stein AD. The Impact of Improving Nutrition During Early Childhood on Education among Guatemalan Adults*. *Econ J* 2009; **119**(537): 734-63.

S27. Chen C-M, Wang Y-Y, Chang S-Y. Effect of in-home fortification of complementary feeding on intellectual development of Chinese children. *Biomed Environ Sci* 2010; **23**(2): 83-91.

S28. Phuka JC, Gladstone M, Maleta K, et al. Developmental outcomes among 18-month-old Malawians after a year of complementary feeding with lipid-based nutrient supplements or corn-soy flour. *Matern Child Nutr* 2012; **8**(2): 239-48.

S29. Pongcharoen T, DiGirolamo AM, Ramakrishnan U, Winichagoon P, Flores R, Martorell R. Long-term effects of iron and zinc supplementation during infancy on cognitive function at 9 y of age in northeast Thai children: a follow-up study. *Am J Clin Nutr* 2011; **93**(3): 636-43.

S30. Nahar B, Hossain M, Hamadani J, et al. Effects of a community-based approach of food and psychosocial stimulation on growth and development of severely malnourished children in Bangladesh: a randomised trial. *Eur J Clin Nutr* 2012; **66**(6): 701-9.

S31. Lozoff B, Castillo M, Clark KM, Smith JB. Iron-fortified vs low-iron infant formula: developmental outcome at 10 years. *Arch Pediatr Adolesc Med* 2012; **166**(3): 208-15.

S32. Gurnida DA, Rowan AM, Idjradinata P, Muchtadi D, Sekarwana N. Association of complex lipids containing gangliosides with cognitive development of 6-month-old infants. *Early Hum Dev* 2012; **88**(8): 595-601.

S33. Manno D, Kowa PK, Bwalya HK, et al. Rich micronutrient fortification of locally produced infant food does not improve mental and motor development of Zambian infants: a randomised controlled trial. *Br J Nutr* 2012; **107**(04): 556-66.

S34. van der Merwe LF, Moore SE, Fulford AJ, et al. Long-chain PUFA supplementation in rural African infants: a randomized controlled trial of effects on gut integrity, growth, and cognitive development. *Am J Clin Nutr* 2013; **97**(1): 45-57.

S35. Mangani C, Cheung YB, Maleta K, et al. Providing lipid-based nutrient supplements does not affect developmental milestones among Malawian children. *Acta Paediatr* 2014; **103**(1): e17-e26.

S36. Surkan PJ, Siegel EH, Patel SA, et al. Effects of zinc and iron supplementation fail to improve motor and language milestone scores of infants and toddlers. *Nutrition* 2013; **29**(3): 542-8.

S37. Singla DR, Shafique S, Zlotkin SH, Aboud FE. A 22-element micronutrient powder benefits language but not cognition in Bangladeshi full-term low-birth-weight children. *J Nutr* 2014; **144**(11): 1803-10.

S38. Yousafzai AK, Rasheed MA, Rizvi A, Armstrong R, Bhutta ZA. Effect of integrated responsive stimulation and nutrition interventions in the Lady Health Worker

programme in Pakistan on child development, growth, and health outcomes: a cluster-randomised factorial effectiveness trial. *Lancet* 2014; **384**(9950): 1282-93.

S39. Yousafzai AK, Obradović J, Rasheed MA, et al. Effects of responsive stimulation and nutrition interventions on children's development and growth at age 4 years in a disadvantaged population in Pakistan: a longitudinal follow-up of a cluster-randomised factorial effectiveness trial. *Lancet Glob Health* 2016; **4**(8): e548-e58.

S40. Locks LM, Manji KP, McDonald CM, et al. The effect of daily zinc and/or multivitamin supplements on early childhood development in Tanzania: results from a randomized controlled trial. *Matern Child Nutr* 2016.

S41. Gardner JMM, Powell CA, Baker-Henningham H, Walker SP, Cole TJ, Grantham-McGregor SM. Zinc supplementation and psychosocial stimulation: effects on the development of undernourished Jamaican children. *Am J Clin Nutr* 2005; **82**(2): 399-405.

S42. Ogunlade AO, Kruger HS, Jerling JC, et al. Point-of-use micronutrient fortification: lessons learned in implementing a preschool-based pilot trial in South Africa. *Int J Food Sci Nutr* 2011; **62**(1): 1-16.

S43. Nga TT, Winichagoon P, Dijkhuizen MA, Khan NC, Wasantwisut E, Wieringa FT. Decreased parasite load and improved cognitive outcomes caused by deworming and consumption of multi-micronutrient fortified biscuits in rural Vietnamese schoolchildren. *Am J Trop Med Hyg* 2011; **85**(2): 333-40.

S44. Murray-Kolb LE, Khatry SK, Katz J, et al. Preschool micronutrient supplementation effects on intellectual and motor function in school-aged nepalese children. *Arch Pediatr Adolesc Med* 2012; **166**(5): 404-10.

S45. Nkhoma OW, Duffy ME, Cory-Slechta DA, et al. Early-stage primary school children attending a school in the Malawian School Feeding Program (SFP) have better reversal learning and lean muscle mass growth than those attending a non-SFP school. *J Nutr* 2013; **143**(8): 1324-30.

S46. Attanasio OP, Fernández C, Fitzsimons EO, Grantham-McGregor SM, Meghir C, Rubio-Codina M. Using the infrastructure of a conditional cash transfer program to deliver a scalable integrated early child development program in Colombia: cluster randomized controlled trial. *BMJ* 2014; **349**: g5785.

S47. Kvestad I, Taneja S, Kumar T, et al. Vitamin B12 and folic acid improve gross motor and problem-solving skills in young north Indian children: A randomized placebo-controlled trial. *PLoS One* 2015; **10**(6): e0129915.
